# Machine learning model for the prediction of pathological complete response after neoadjuvant chemotherapy in breast cancer

**DOI:** 10.1186/s12885-026-17115-7

**Published:** 2026-10-08

**Authors:** Eva Reisig, Lie Cai, Maria Müller, Benedikt Schäfgen, André Pfob

**Affiliations:** 1 Breast Unit, Department of Obstetrics and Gynecology, Heidelberg University Hospital, Heidelberg, Germany; 2 Department of Obstetrics and Gynecology, Münster University Hospital, Münster, Germany

**Keywords:** Breast cancer, Machine learning, Neoadjuvant chemotherapy, Pathological response, Mammography, Ultrasound

## Abstract

**Background:**

Breast cancer patients receiving neoadjuvant treatment may benefit from a presurgical assessment of complete response to avoid unnecessary surgeries. We aimed to develop a machine learning model that predicts pathological complete response (pCR) based on results from different image modalities as well as tumor and patient characteristics.

**Methods:**

Clinical data from 388 cases of invasive breast cancer receiving neoadjuvant treatment were extracted in this retrospective, monocentric study. Findings from ultrasound, mammography and magnetic resonance imaging combined with patient and histopathological information were included. A Pearson Product-Moment correlation and a LASSO regularization were performed to preselect the most relevant features for the outcome. Based on the preselected variables, we developed and validated a machine learning algorithm (logistic regression with elastic net penalty (GLM)) to predict pCR. The model’s performance was evaluated via area under the curve (AUROC), false positive rate (FPR) and positive predictive value (PPV).

**Results:**

In the total cohort, pCR was detected in 158 out of 388 cases, resulting in a pCR rate of 40.7%. From 111 collected variables, 8 variables were removed due to a high correlation with other features. After the LASSO regularization, 14 variables were included as predictors for the final model. In the validation set, the GLM achieved an AUROC of 0.76 with a FPR of 8.7% and a PPV of 75%.

**Conclusions:**

Through the combination of imaging and clinicopathological features, our machine learning model shows a promising approach to improve the prediction of pCR in breast cancer patients receiving neoadjuvant treatment. In combination with the LASSO regularization, it allows interesting insights into the predictive potential of different variables, which are available as part of standard clinical practice.

**Trial registration:**

Not applicable.

**Supplementary Information:**

The online version contains supplementary material available at https://doi.org/10.1186/s12885-026-17115-7.

## Background

Patients with inflammatory, locally advanced, or unresectable breast cancer, as well as those with high-risk HER2-positive or triple-negative subtypes, receive neoadjuvant chemotherapy as part of standard treatment [1, 2]. Originally indicated for inoperable or locally advanced tumors, neoadjuvant therapy has subsequently been shown to increase the feasibility of breast-conserving surgery [1]. Despite achieving clinical complete response (cCR), surgical resection remains necessary for all patients to allow histopathological confirmation of residual disease [2, 3]. Reliable preoperative identification of pathological complete response (pCR) would therefore enable the avoidance of unnecessary surgical procedures.

During chemotherapy, patients are routinely assessed clinically and often undergo imaging for follow-up, although no standardized guidelines currently exist regarding its implementation [1, 3]. The 2021 ASCO guideline recommends using the imaging modality that was most informative at baseline —mammography, ultrasound, or magnetic resonance imaging (MRI)— for follow-up [1]. This results in a substantial amount of heterogeneous imaging data, which could be leveraged for pCR prediction. Machine learning offers a promising approach to integrating this multimodal data, as it can manage large numbers of variables and accommodate missing values arising from irregular imaging.

Several studies have examined the implementation of artificial intelligence (AI) for predicting response to neoadjuvant chemotherapy in breast cancer patients. Li et al. published a meta-analysis, which focused on ultrasound-based radiomics for early response prediction [4], while Valizadeh et al. examined the results from ultrasound-based machine learning models [5]. However, the included studies focused on extracting features from the images directly and did not include mammography or MRI. Several studies have constructed MRI-based models to predict pCR [6–8], while MRI is not part of the conventional diagnostics for breast cancer and therefore not implemented routinely [2].Other studies have focused on only clinicopathological features for developing machine learning models [9–12].

For optimal generalizability and transferability to clinical practice, it would be desirable to create a model which includes results from all image modalities that are conducted until surgery as well as clinical and tumor characteristics. The goal of the present study is to examine whether a machine learning model can reliably predict pCR on new data to avoid unnecessary surgeries in breast cancer patients, and whether it profits from incorporating the overall clinical context in its analysis.

## Methods

### Patient recruitment

We used data from 500 patients with 506 breast cancers, who were treated at the Breast Centre of the University Hospital of Heidelberg between 2019 and 2022. Included patients were adults and underwent neoadjuvant chemotherapy followed by either breast-conserving therapy (BCT) (*n* = 222), mastectomy (*n* = 160), or both (*n* = 6). The exclusion criteria are as follows:

1. ex domo therapy (*n* = 32);
2. clinical stage cT4 or ycT4 breast cancer (*n* = 28);
3. history of ipsilateral breast cancer (*n* = 23);
4. absence of surgical therapy due to palliative situation (*n* = 9);
5. Cancer of Unknown Primary (*n* = 7).
6. loss to follow up, patient refusal of neoadjuvant chemotherapy, or incomplete image findings (*n* = 19).

For our final machine learning model, 388 cases were included.

### Collected variables

Patient characteristics were collected. All patients received an initial breast biopsy and were treated with neoadjuvant treatment (NACT) following the standard protocols of our breast centre being grounded on national guidelines [2]. Results from clinical examination, ultrasound (US), mammography (MAMMO) and MRI at the time of preNACT, NACT1, NACT2 and postNACT were collected. All procedures were performed in everyday clinical practice. 2D mammography and digital breast tomosynthesis images were acquired using the Mammomat Inspiration (Siemens AG, Erlangen, Germany). Breast MRI was performed using a 1.5-tesla field scanner (Avanto; Siemens Medical, Erlangen, Germany) with a bilateral breast coil (Seven-Channel Biopsy Breast Array; Invivo, Orlando, FL, USA). Images were evaluated by breast imaging specialists (500 MRIs per year), who selected DCE images based on in-house standardized protocols.

A large dataset was retrospectively created based on information from discharge letters and the ultrasound, mammography, MRI and histopathology reports. Image-based information on tumor size, multifocality, multicentricity, suspect lymph nodes, microcalcifications and clinical remission status were taken from the radiologists’ descriptions. After NACT, we collected ycT and ycN stages from the reports and summarized the clinical remission status in separate imaging assessment variables as described below.

Currently, there is no standardized definition of clinical complete response. As described in our previous study [13], imaging results after NACT were categorized in three groups, either cCR with clear imaging, non cCR with the detection of residual tumor or near-cCR with minimal signs of residual tumor (e.g. architectural distortion or residual microcalcifications). The near cCR category included imaging, which appeared suspect, but did not show a clearly delineable lesion. This category was incorporated into the ycT stages of the different image modalities with an additional ycTnear label. In some cases, a clear description of the imaging findings was missing in the radiological reports. For this reason, a retrospective categorization of these cases was not possible, and they were labelled as ycTX. Consequently, the imaging assessment variables summarize the ycT stages from each modality with the categories cCR (ycT0), non-cCR (ycTis – ycT4) or near-cCR (ycTnear). The ycTX label was regarded as a missing value for the assessment variables.

Information from 111 variables was used for the analysis. More detailed descriptions of the variables are available in supplement table S1.

The results from the histopathological examination of the surgical resection were used to assess pCR as the outcome variable. In our study, pCR was defined as the absence of invasive and non-invasive tumor cells in the histopathology specimen.

### Model preparation

Our study closely followed the machine learning guidelines previously published by our group [14]. For data preparation, variables were converted into either categorial or numeric variables. Variables with 50% or more of missing values were removed. We randomly split the cohort into a development set (*n* = 311, 80%) and a validation set (*n* = 77, 20%).

Before training the final machine learning model, we selected the most relevant variables from the dataset. A Pearson Product-Moment correlation was performed on the development set to remove numeric variables that had a higher correlation than 90% with other variables. The remaining features were pre-processed using the recipe shown in the data supplement including steps such as K-nearest neighbour with 5 neighbours, rare category grouping and one-hot encoding. Then, we applied a Least Absolute Shrinkage and Selection Operator (LASSO) regularization to extract the most relevant features from the dataset.

Using only the variables selected by the LASSO regularization, we built a logistic regression with elastic penalty (GLM) as the final model. For one hot encoded variables, we included all categories even if only one of them was chosen by the LASSO regularization. The GLM-model was trained with a grid hyperparameter search and internally validated using tenfold cross-validation with 3 repeats. As a supplementary analysis for performance comparison, three other machine-learning models were trained, namely Extreme-Gradient Boosting tree (XGBoost), Multivariate Adaptive Regression Spline (MARS) and a Support Vector Machine (SVM) with polynomial kernel. These models used the same variables and preprocessing steps as the GLM and were trained analogously, with grid search, tenfold cross-validation and 3 repeats.

For the GLM model, variable importance was calculated using the varImp function, which takes the absolute value of the model’s coefficients and scales them from 0 to 100. For each categorical variable, the mean importance score from all indicator variables was calculated. Odds ratios were determined for all included variables with bootstrapped 95% confidence intervals (2000 replications).

We used R version 4.5.1 for all analysis.

### Statistical analysis

We calculated the AUROC for the development and the validation set. As the focus of this study was to find patients with no residual tumor, pCR patients were set as positive cases. The false positive rate (FPR) and the positive predictive value (PPV) were calculated so that we could analyse the model’s capacity to reliably exclude residual diseases. Spiegelhalter’s Z and Brier Score were calculated [36], and calibration plots were created to evaluate the model’s calibration. McNemar’s test was applied for comparison between predictive performance, and AUROC values were compared with bootstrap replicates.

For this study, we considered values of *P* < 0.05 as significant.

## Results

### Clinical characteristics

In this study, data from 388 breast cancers were analysed, with an average patient age of 49.4 years at the time of diagnosis. Table 1 shows the distribution of the baseline clinical characteristics including the distribution of all input variables for the final model. Only one significant difference between the development set and the validation set could be found, which was the distribution of the ycT1c category (from the US/MAMMO/MRI ycT stage) with a significantly higher proportion in the validation set (35.1% (27/77) v 22.2% (69/311), *p* = 0.019). Pathological complete response (pCR) after neoadjuvant chemotherapy was diagnosed in 158 cases (40,7%). In the development set, the pCR rate was at 40,8% (127 from 311 cases) and in the validation set at 40,3% (31 from 77 cases).

**Table 1:** Baseline clinical characteristics and variable comparison between the development set and validation set

Characteristic	Whole Cohort (*n* = 388)	Development Set (*n* = 311)	Validation Set (*n* = 77)	*P*
Age (SD)	49.4 (11.9)	49.5 (11.9)	49.1 (12.0)	0.816
Affected breast, No. (%)				0.524
Left	194 (50.0)	153 (49.2)	41 (53.2)	
Right	194 (50.0)	158 (50.8)	36 (46.8)	
Menopausal status, No. (%)				
Premenopausal	183 (48.2)	145 (47.4)	38 (50.0)	0.719
Perimenopausal	29 (7.6)	25 (8.2)	4 (5.3)	0.385
Postmenopausal	168 (44.2)	134 (44.1)	34 (44.7)	0.918
Missing	8	7	1	
cT, No. (%)				
T1b	9 (2.3)	9 (2.9)	0	0.131
T1c	105 (27.1)	83 (26.7)	22 (28.6)	0.739
T2	236 (60.8)	188 (60.5)	48 (62.3)	0.761
T3	38 (9.8)	31 (10.0)	7 (9.1)	0.817
cN, No. (%)				
cN0	175 (45.1)	137 (44.1)	38 (49.4)	0.403
pN0	50 (12.9)	42 (13.5)	8 (10.4)	0.465
cN+	23 (5.9)	18 (5.8)	5 (6.5)	0.814
pN+	138 (35.6)	113 (36.3)	25 (32.5)	0.614
cNx	2 (0.5)	1 (0.3)	1 (1.3)	0.284
Carcinoma in situ associated, No. (%)				
none	303 (78.1)	242 (77.8)	61 (79.2)	0.789
DCIS	82 (21.1)	66 (21.2)	16 (20.8)	0.932
LCIS	2 (0.5)	2 (0.6)	0	0.480
DCIS + LCIS	1 (0.3)	1 (0.3)	0	0.618
Grading, No. (%)				
I	3 (0.8)	3 (1.0)	0	0.386
II	145 (37.5)	121 (39.0)	24 (31.2)	0.202
III	238 (61.5)	185 (59.7)	53 (68.8)	0.140
IV	1 (0.3)	1 (0.3)	0	0.618
Missing	1	1	0	
ER, No. (%)				
Positive	197 (50.3)	157 (50.5)	40 (51.9)	0.818
Negative	178 (45.9)	142 (45.7)	36 (46.8)	0.863
Weakly positive	13 (3.4)	12 (3.9)	1 (1.3)	0.264
PR, No. (%)				
Positive	159 (41.0)	125 (40.2)	34 (44.2)	0.527
Negative	214 (55.2)	173 (55.6)	41 (53.2)	0.707
Weakly positive	15 (3.9)	13 (4.2)	2 (2.6)	0.519
HER2, No. (%)				0.050
Positive	105 (27.1)	91 (29.3)	14 (18.2)	
Negative	283 (72.9)	220 (70.7)	63 (81.8)	
KI-67 (SD)	52.9 (23.9)	52.4 (23.6)	54.8 (25.3)	0.127
Missing	1	1	0	
Tumor biology subgroup, No. (%)				
TNBC	147 (37.9)	112 (36.0)	35 (45.5)	0.127
HER2-/HR+	136 (35.1)	108 (34.7)	28 (36.4)	0.787
HER2+	105 (27.1)	91 (29.3)	14 (18.2)	0.050
Breast density ACR, No. (%)				
I	30 (7.9)	27 (8.8)	3 (3.9)	0.157
II	161 (42.1)	125 (40.8)	36 (47.4)	0.303
III	148 (38.7)	122 (39.9)	26 (34.2)	0.365
IV	43 (11.3)	32 (10.5)	11 (14.5)	0.321
Missing	6	5	1	
US multifocality preNACT, No. (%)				0.363
Yes	102 (26.5)	85 (27.5)	17 (22.4)	
No	283 (73.5)	224 (72.5)	59 (77.6)	
Missing	3	2	1	
US largest DIA NACT1 in mm (SD)	14.7 (9.6)	14.8 (9.8)	14.0 (9.1)	0.486
Missing	23	17	6	
MAMMO largest DIA NACT1 in mm (SD)	16.1 (14.2)	15.8 (14.2)	16.9 (14.3)	0.608
Missing	127	105	22	
Palpability postNACT, No. (%)				0.835
Yes	72 (18.7)	57 (18.4)	15 (19.5)	
No	314 (81.3)	252 (81.6)	62 (80.5)	
Missing	2	2	0	
US assessment postNACT, No. (%)				
cCR	150 (38.9)	117 (37.9)	33 (42.9)	0.421
Near-cCR	67 (17.4)	59 (19.1)	8 (10.4)	0.071
Non-cCR	169 (43.8)	133 (43.0)	36 (46.8)	0.557
Missing	2	2	0	
Mammo assessment postNACT, No. (%)				
cCR	129 (40.8)	99 (40.1)	30 (43.5)	0.612
Near-cCR	68 (21.5)	58 (23.5)	10 (14.5)	0.108
Non-cCR	119 (37.7)	90 (36.4)	29 (42.0)	0.397
Missing	72	64	8	
US/MAMMO/MRI ycT stage, No. (%)				
ycT0 (cCR)	86 (22.2)	68 (21.9)	18 (23.4)	0.775
ycT1a	9 (2.3)	6 (1.9)	3 (3.9)	0.305
ycT1b	46 (11.9)	39 (12.5)	7 (9.1)	0.402
ycT1c	96 (24.7)	69 (22.2)	27 (35.1)	0.019
ycT2	33 (8.5)	28 (9.0)	5 (6.5)	0.450
ycT3	6 (1.5)	6 (1.9)	0 (0.0)	0.219
ycTis	21 (5.4)	16 (5.1)	5 (6.5)	0.640
ycTnear	79 (20.4)	69 (22.2)	10 (13.0)	0.073
ycTX	12 (3.1)	10 (3.2)	2 (2.6)	0.779
Outcome, No. (%)				0.927
pCR	158 (40.7)	127 (40.8)	31 (40.3)	
Non-pCR	230 (59.3)	184 (59.2)	46 (59.7)	

Legend: P-values were calculated using chi-square test for categorical variables and t-test for continuous variables

SD, standard deviation; DCIS, ductal carcinoma in situ; LCIS, lobular carcinoma in situ; PR, progesterone receptor; ER, estrogen receptor; HER2, human epidermal growth factor receptor 2; TNBC, triple-negative breast cancer; HR, hormone receptor; cCR, clinical complete response; pCR, pathological complete response

### Features

**Fig. 1:**
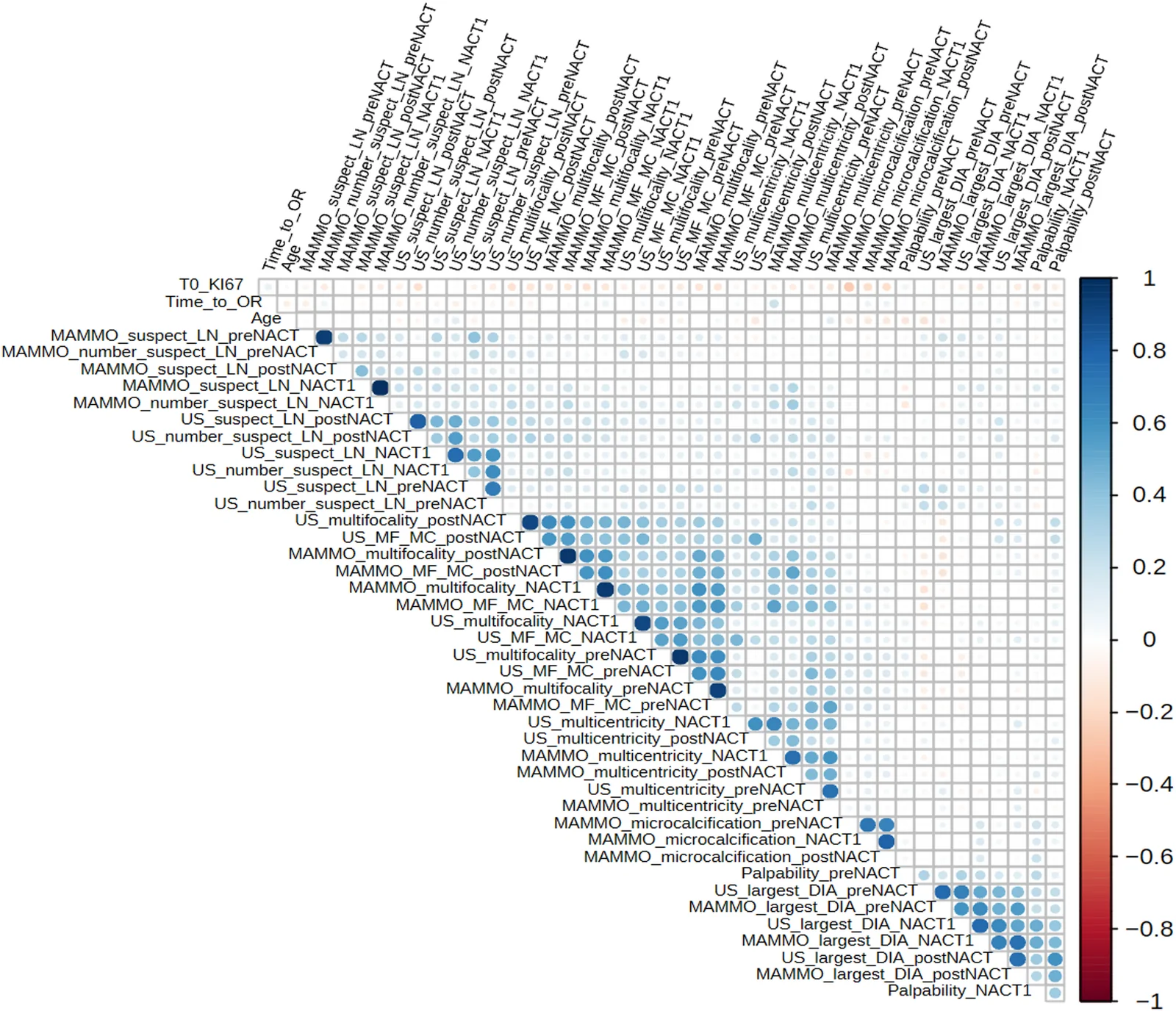
Pearson correlation coefficient matrix plot. Color illustrates the sign and strength of the correlation, while spot size is proportional to its absolute value

A detailed description of the initial 111 variables can be found in supplement table S1. 41 of the variables had to be removed due to 50% or more of missing values. Figure 1 shows the heatmap of the Pearson Correlation Coefficient Matrix (PCCM). The following 8 variables were removed:

MAMMO multifocality/multicentricity preNACT, US multifocality/multicentricity preNACT, MAMMO multifocality/multicentricity NACT1, US multifocality/multicentricity NACT1, MAMMO number of suspect lymph nodes NACT1, MAMMO multifocality/multicentricity postNACT, US multifocality postNACT, MAMMO number of suspect lymph nodes preNACT.

The LASSO Feature Selection selected the following 14 variables, which were included in the final model:

US multifocality preNACT, US largest DIA NACT1, MAMMO largest DIA NACT1, Palpability postNACT, Menopause, Breast affected, cN stage, T0 estrogen receptor (ER) status, T0 progesterone receptor (PR) status, Tumor biology subgroup, Breast Density (American College of Radiology [ACR] categories), US assessment postNACT, MAMMO assessment postNACT, US/MAMMO/MRI ycT stage.

### Model performance

**Fig. 2:**
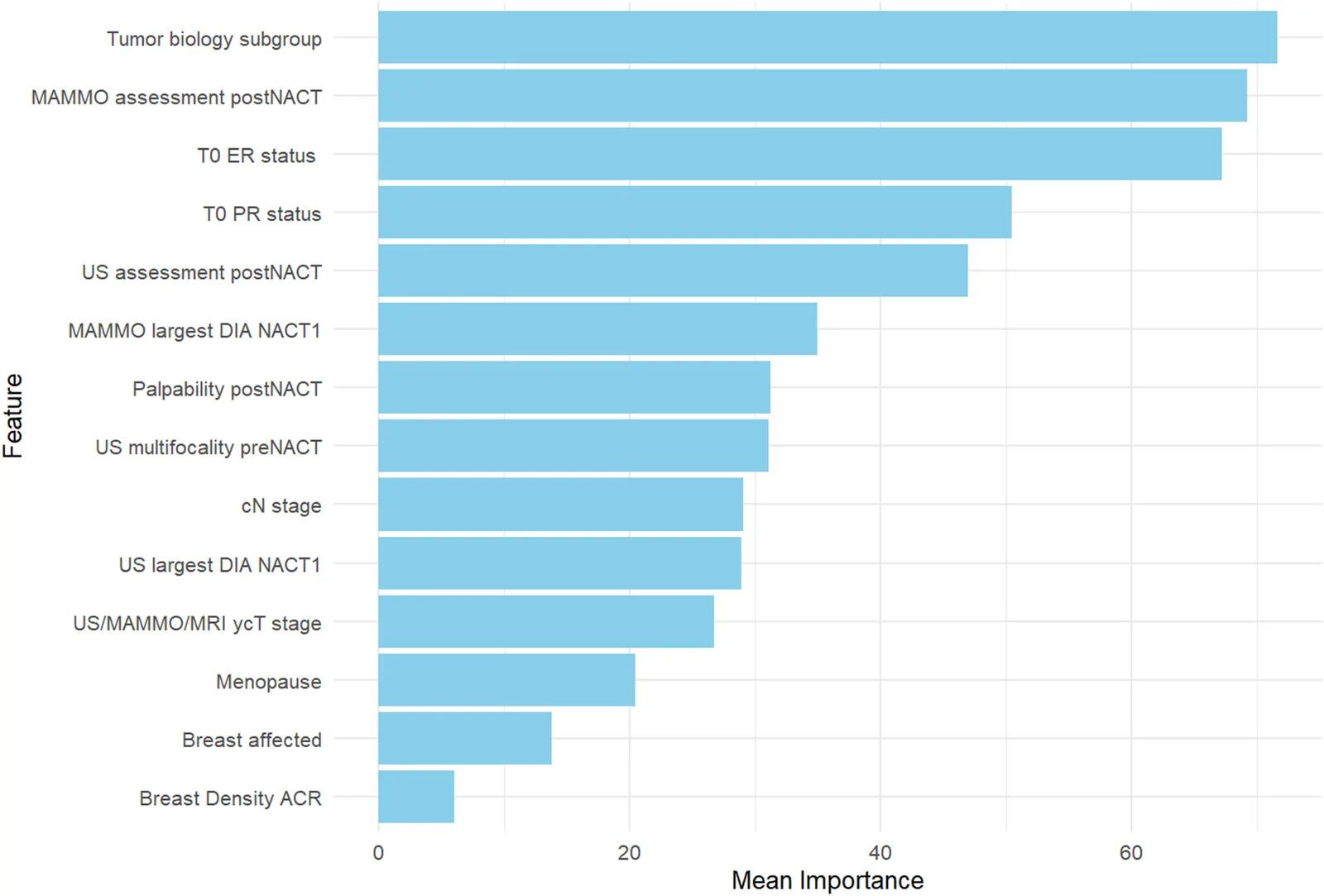
Feature importance of the GLM using the varImp function. Importance scores were scaled between 0 and 100. For categorical variables, the mean importance score from their indicator variables is represented

**Fig. 3:**
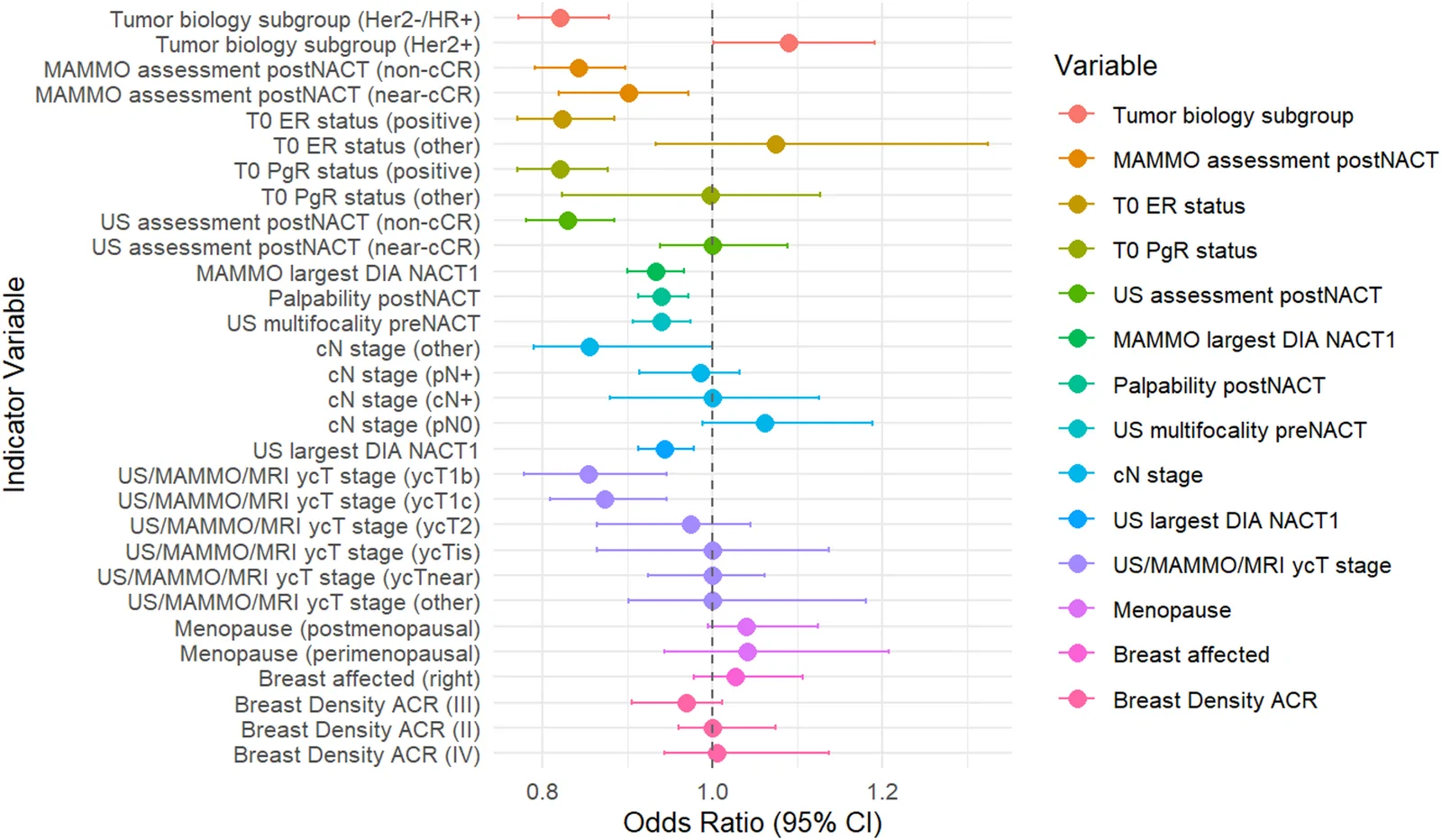
Odds ratios of the features included in the GLM. Odds ratios were calculated for the included indicator variables. As part of the preprocessing recipe, the “other” category grouped rare categories occurring in less than 5% of cases. Due to one-hot encoding, each categorical variable is represented by one fewer indicator variable than actual categories. Variables are ordered by the mean odds ratio of their categories and are illustrated with 95% confidence intervals

Figure 2 illustrates the feature importance of the GLM, while Fig. 3 represents the odds ratios of each included variable. The five most important variables became:

Tumor biology subgroup, MAMMO assessment postNACT, T0 ER status, T0 PR status and US assessment postNACT.

**Fig. 4:**
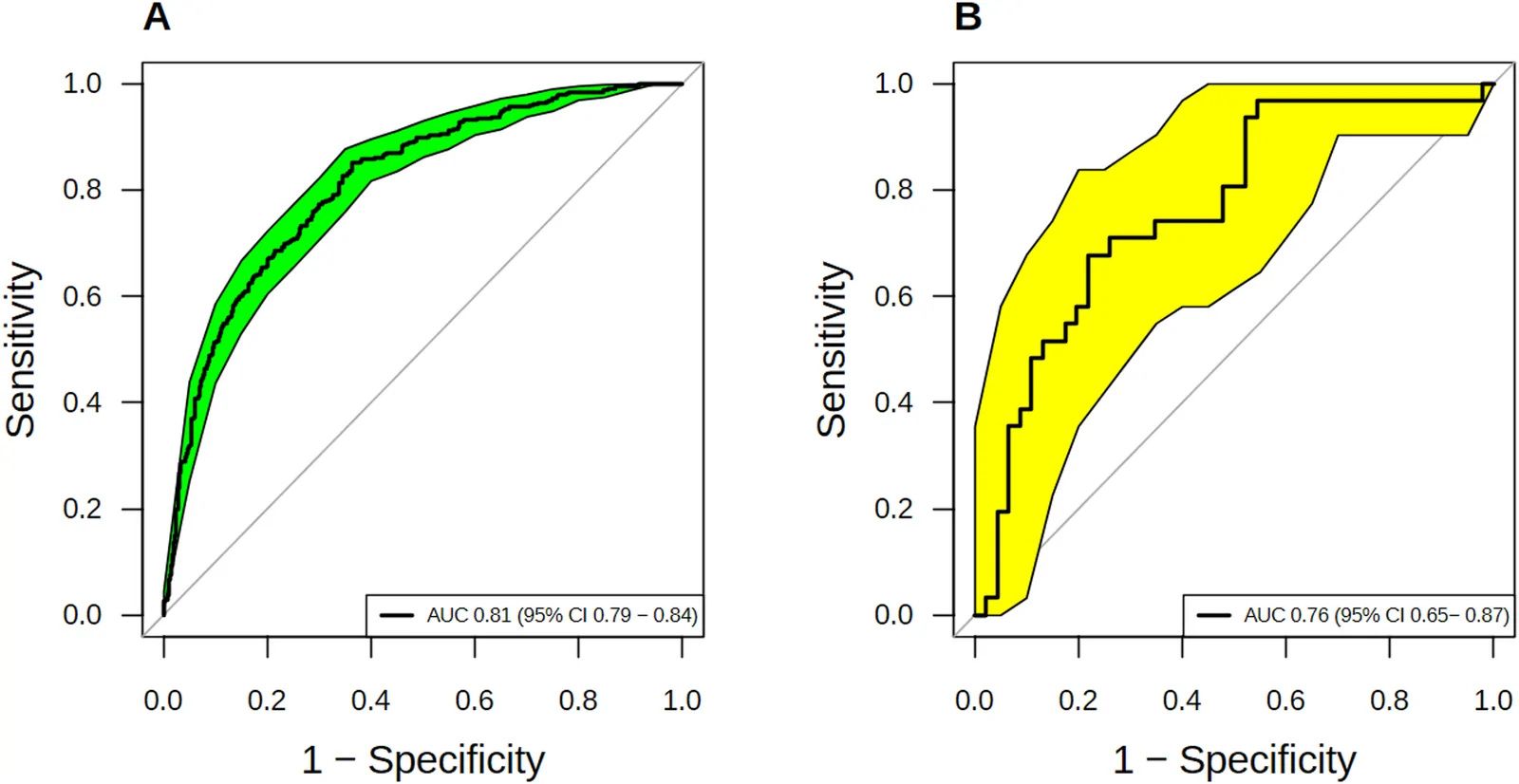
**A** ROC curve of the GLM model with standard deviation in the development set. **B** ROC curve of the GLM model with standard deviation in the validation set

Figure 4 shows the receiver operating characteristic curves (ROC) of the GLM in the development and validation set. The model achieved an AUC of 0.81 (95% CI 0.79–0.84) in the development set. In the validation set, the AUC was at 0.76 (95% CI 0.65–0.87).

In Table 2, the performance metrics of the GLM in the development and validation set are illustrated. The internal validation includes 933 cases as the model was trained with three repeats offering the results of 311 times 3 cases. In the development set, the GLM achieved a FPR of 4.9% and a PPV of 81,8%. While validating, the FPR was at 8.7% and the PPV at 75% with an accuracy of 70.1%, which is significantly higher than the no information rate (59,7%).

**Table 2:** Performance metrics of the GLM in the development and validation set

Performance Metric	Development Set	Validation Set
False Positive Rate	4.9% (27 of 552)	8.7% (4 of 46)
Positive Predictive Value	81.8% (121 of 148)	75.0% (12 of 16)
Accuracy (ACC)	69.2%	70.1%
No Information Rate (NIR)	59.2%	59.7%
P-Value (ACC > NIR)	< 0.001	0.039

The data supplement lists the performance metrics of the additional models for comparison. In the validation set, the GLM achieved a descriptively lower FPR (8.7% (4 of 46) v 10.9% (5 of 46), 10.9% (5 of 46) and 10.9% (5 of 46)) and a higher PPV (75.0% (12 of 16) v 66.7% (10 of 15), 68.8% (11 of 16) and 66.7% (10 of 15)) than the XGboost, MARS algorithm and SVM. No significant difference in model performance (*p* = 1.00) or AUC (0.76 v 0.79, 0.80 and 0.77; *p* = 0.34, *p* = 0.29 and *p* = 0.79) was observed in comparison to the three algorithms.

**Fig. 5:**
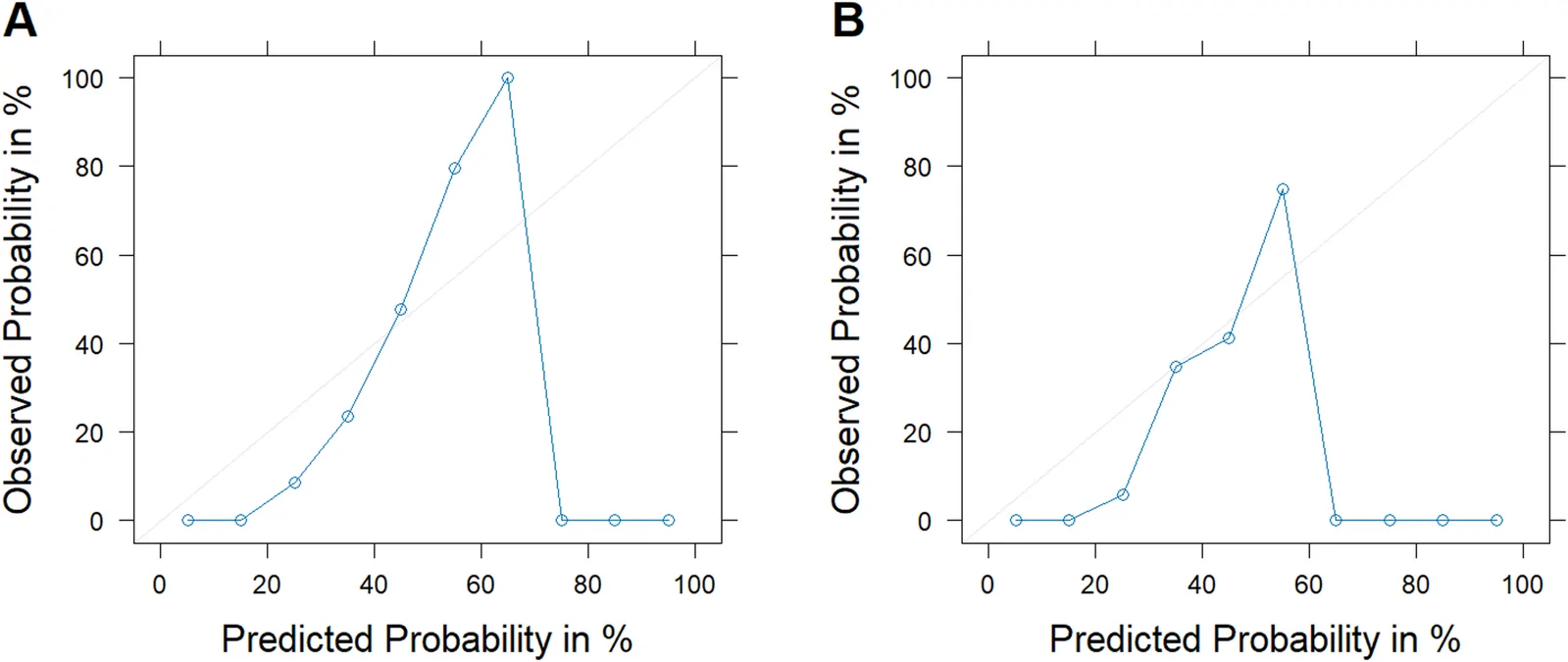
**A** Calibration plot of the GLM model in the development set. **B** Calibration plot of the GLM model in the validation set

Figure 5 shows the calibration plots of the model. The Spiegelhalter’s test showed a good calibration of the model in the validation set with a Z-value of -1.72 (*p* = 0.086). The Brier score was at 0.20.

## Discussion

In this study, we created a GLM model for the prediction of pCR after neoadjuvant chemotherapy in patients with breast cancer. The model was trained with features from different image modalities and clinicopathology, which were preselected by a LASSO feature selection. The model achieved an AUC of 0.76 with a FPR of 8.7% and a PPV of 75% in the validation set.

PCR prediction in breast cancer patients has been an active area of research, with many studies on radiomics- and deep learning-based models [15–18]. While these approaches extract features beyond what the human eye can detect, this comes at the cost of increased complexity in clinical application, e.g. by requiring standardized image acquisition on similar hardware to ensure reproducibility [19]. We chose a simpler logistic regression model relying on predefined, clinical interpretable variables from medical reports. Our model showed a descriptively lower FPR and higher PPV than more complex algorithms (XGboost, MARS and SVM). Other studies have used logistic regression to investigate the predictive power of image derived parameters, such as MRI based functional tumor volume (FTV) [20–22]. Moreover, BI-RADS descriptors from ultrasounds were combined with clinicopathology to create a prediction model based on a relatively small study cohort. It achieved an AUC value of 0.72 in an unseen validation set [23]. Evidence on conventional prediction models incorporating all three modalities remains limited. There was a study investigating the predictive power of baseline imaging features from ultrasound, mammography and MRI combined with pathological features. However, no validation set was included. They found that mammography at baseline did not provide additional information to ultrasound, MRI and pathology [24]. In contrast, postNACT mammography became the second most important feature for our model, indicating the diagnostic utility of mammography. Another logistic model with multimodal imaging features and clinicopathology achieved an AUC of 0.75 in an unseen validation set [25], which is comparable to the AUC of 0.76 in our study.

Other studies have evaluated the diagnostic performance of mammography, breast ultrasound and MRI as interpreted by clinicians. The results vary strongly, however there is a common opinion that, with imaging alone, clinicians cannot reliably exclude residual disease [26–29]. This was confirmed in a previous analysis of the study data [13]. When setting pCR as positive cases, a FPR of 26.6% for ultrasound alone and 19.7% for mammography alone was calculated. The combination of both modalities resulted in a much lower FPR of 11% and a PPV of 71.6%. Our machine learning algorithm could achieve slightly better results by considering the overall clinical context of the patients. Whereas the initial evaluation was a strictly retrospective description of the data, the machine learning model was able to perform on an unseen validation set with a lower FPR, a higher PPV and an acceptable AUC value of 0.76.

It remains unclear which rate of missed cancer is acceptable to omit surgery based on pCR prediction. When deescalating to sentinel lymph node biopsy, a missed-cancer rate of up to 10% has shown no impairment of survival or regional control [30, 31]. This threshold was achieved by our cohort and was also targeted in a diagnostic trial evaluating vacuum-assisted biopsy for pCR prediction [32]. In contrast, another study analysing the value of tumor-bed biopsies required a predictive value of 90% for diagnosed pCR, a threshold that was not reached in our dataset [33].

A strength of our study is the retrospective collection of information from examination reports and discharge letters of patients receiving standard treatment, enabling routine clinical application. However, combination with additional methods should be examined to increase predictive performance prior to treatment guidance. Particularly, addition of postNACT vacuum-assisted biopsies should be further explored, as these have shown promising results in multivariate AI evaluation and constitute a less invasive method than surgery [34].

The tumor biology subgroup, the estrogen receptor status and the progesterone receptor status are among the four most important features of our model. This corresponds to the different pCR rates of the tumor subgroups, which are higher among triple negative and HER2-positive/HR-negative subgroups [35]. Our machine learning model considers the impact of this in its evaluation. Moreover, it attached great importance to the assessment of mammography and ultrasound postNACT. However, other clinical variables can only slightly contribute to the prediction of pCR.

The largest diameter of the tumor as seen in mammography NACT1 became the sixth most important variable. While this variable should indicate the tumor’s initial sensitivity to chemotherapy, it might also play an essential role for the near-cCR group, where the imaging appears suspect but does not show a completely delineable lesion. These cases are particularly difficult for clinicians to interpret. In the previous analysis, the FPR increased by 23.8% points to 34.8% when including near-cCR in the definition of cCR [13]. Machine learning models have the capacity to consider many variables at once and can therefore try to find additional predictors for more diagnostically ambiguous cases.

Our study also shows some limitations. The GLM model moderately improved the results from clinic-based imaging assessment; however, it did not show a substantially better performance. This might be due to the moderate sample size in comparison to the large number of analysed variables. We sought to address this issue by implementing a LASSO feature selection and only including relevant variables in the final model training. Yet, a larger cohort might yield more precise predictions. Another limitation of our study is the retrospective, monocentric design. Our GLM model was validated on an unseen dataset using a random split. Nonetheless, future validation in an external set, optimally in a prospective cohort, must be performed. Furthermore, we analysed imaging assessments from different timepoints, which may possibly create dependency between variables. However, we excluded high-correlation variables through the application of a PCCM, while a LASSO regularization selected relevant features. In the final models, no duplicate variables assessed at different time points were included.

## Conclusions

A machine learning model analysing clinical and imaging data of breast cancer patients can accurately predict pathological complete response after neoadjuvant treatment. By facilitating the interpretation of routinely collected clinical variables, our model may help reduce unnecessary surgical interventions in cases of pCR.

## Supplementary information

Below is the link to the electronic supplementary material.

Supplementary Material 1

## Data Availability

Data available from the corresponding author upon reasonable request.

## Abbreviations

pCR: Pathological complete response
GLM: Logistic regression with elastic net penalty
AUROC: Area under the curve
FPR: False positive rate
PPV: Positive predictive value
cCR: Clinical complete response
AI: Artificial intelligence
MRI: Magnetic resonance imaging
BCT: Breast-conserving therapy
NACT: Neoadjuvant treatment
US: Ultrasound
MAMMO: Mammography
LASSO: Least Absolute Shrinkage and Selection Operator
PCCM: Pearson Correlation Coefficient Matrix
ER: Estrogen receptor
PR: Progesterone receptor
ACR: American Society of Radiology
ROC: Receiver operating characteristic curve
ACC: Accuracy
NIR: No information rate
HR: Hormone receptor
FTV: Functional tumor volume
