# Supplementary material for: Machine learning model for the prediction of pathological complete response after neoadjuvant chemotherapy in breast cancer
Source: BMC Cancer. 2026 Oct 8;26:1229. doi: 10.1186/s12885-026-17115-7 (PMC13670700; doi:10.1186/s12885-026-17115-7)
Supplement: Supplementary file 1 [file 12885_2026_17115_MOESM1_ESM.docx]

Supplement for

Machine learning model for the prediction of Pathological Complete Response after Neoadjuvant Chemotherapy in Breast Cancer

We provide:

1. A description table of the variables.
2. A description of the pre-processing recipe.
3. The performance metrics of three additional machine learning algorithms.
1) Table S1. Description table of the variables.

A table with the names of all variables that were considered for the model building. For each variable, the table shows the labels, an explanation and the exclusion reason or information about inclusion.

| Variable | Labels | Explanation | Inclusion/removal (reason for removal) |
| --- | --- | --- | --- |
| Age |  | Age at time of diagnosis | Removed (LASSO) |
| Breast affected | Left | Bilateral breast cancers are captured seperately | Included |
|  | Right |  |  |
| Menopause | Premenopausal |  | Included |
|  | Perimenopausal |  |  |
|  | Postmenopausal |  |  |
| cT stage | cT0 | cT stage, TNM classification (before NACT) | Removed (LASSO) |
|  | cTis |  |  |
|  | cT1mi |  |  |
|  | cT1a |  |  |
|  | cT1b |  |  |
|  | cT1c |  |  |
|  | cT2 |  |  |
|  | cT3 |  |  |
|  | cT4a |  |  |
|  | cT4b |  |  |
|  | cT4c |  |  |
|  | cT4d |  |  |
| cN stage | cN0 | No clinically suspicious lymph nodes | Included |
|  | pN0 | Clinically suspicious lymph nodes with benign histology |  |
|  | cN+ | Clinically suspicious lymph nodes without histological confirmation |  |
|  | pN+ | Histologically confirmed lymph node metastasis |  |
|  | cNX | Unclear lymph node status |  |
| T0 associated carcinoma in situ | None | Presence of an associated carcinoma | Removed (LASSO) |
|  | DCIS (Ductal carcinoma in situ) |  |  |
|  | LCIS (Lobular carcinoma in situ) |  |  |
|  | DCIS and LCIS |  |  |
| T0 grading | I | Histological grading (before NACT) | Removed (LASSO) |
|  | II |  |  |
|  | III |  |  |
|  | IV |  |  |
| T0 histological subtype | Invasive ductal carcinoma (NST) | Histological subtype (before NACT) | Removed (LASSO) |
|  | Invasive lobular carcinoma |  |  |
|  | Medullary carcinoma |  |  |
|  | Mucinous carcinoma |  |  |
|  | NST plus invasive lobular carcinoma |  |  |
|  | NST plus special type |  |  |
|  | Apocrine carcinoma |  |  |
|  | Invasive micropapillary carcinoma |  |  |
|  | Metaplastic carcinoma |  |  |
|  | Other |  |  |
| T0 ER status | Negative | Estrogen receptor status: Positive when ≥ 10% positive tumor cells, weakly positive when 1-9% positive tumor cells, negative when < 1% positive tumor cells | Included |
|  | Positive |  |  |
|  | Weakly positive |  |  |
| T0 PR status | Negative | Progesterone receptor status: Positive when ≥ 10% positive tumor cells, weakly positive when 1-9% positive tumor cells, negative when < 1% positive tumor cells | Included |
|  | Positive |  |  |
|  | Weakly positive |  |  |
| T0 HER2 status | 0 | HER2 status using immunohistochemistry and fluorescence in situ hybridization (FISH) or chromogenic in situ hybridization (CISH) | Removed (LASSO) |
|  | 1+ |  |  |
|  | 2+ with FISH/CISH negative |  |  |
|  | 2+ with FISH/CISH positive |  |  |
|  | 3+ |  |  |
| T0 HER2 assessment | Negative | HER2 assessment based on T0 HER2 status: positive for 2+ with FISH/CISH positive or 3+ | Removed (LASSO) |
|  | Positive |  |  |
| T0 KI-67 |  | KI-67 index in % | Removed (LASSO) |
| Tumor biology subgroup | TNBC | Combined tumor biology subgroup, weakly positive HR cases are considered as TNBC or HER2 positive | Included |
|  | HER2 negative / HR positive |  |  |
|  | HER2 positive |  |  |
|  | Unassigned |  |  |
| Palpability preNACT | No | Palpability in physical examination before NACT | Removed (LASSO) |
|  | Yes |  |  |
| US largest DIA preNACT |  | Largest diameter of the tumor in mm, evaluated by ultrasound before NACT | Removed (LASSO) |
| US multifocality preNACT | No | Presence of multifocality, evaluated by ultrasound before NACT | Included |
|  | Yes |  |  |
| US multicentricity preNACT | No | Presence of multicentricity, evaluated by ultrasound before NACT | Removed (LASSO) |
|  | Yes |  |  |
| US MF/MC preNACT | No | Presence of multifocality or multicentricity, evaluated by ultrasound before NACT | Removed (high correlation) |
|  | Yes |  |  |
| US suspect LN preNACT | No | Presence of suspicious lymph nodes, evaluated by ultrasound before NACT | Removed (LASSO) |
|  | Yes |  |  |
| US number suspect LN preNACT |  | Number of suspect lymph nodes, evaluated by ultrasound before NACT | Removed (LASSO) |
| MAMMO largest DIA preNACT |  | Largest diameter of the tumor in mm, evaluated by mammography before NACT | Removed (LASSO) |
| MAMMO multifocality preNACT | No | Presence of multifocality, evaluated by mammography before NACT | Removed (LASSO) |
|  | Yes |  |  |
| MAMMO multicentricity preNACT | No | Presence of multicentricity, evaluated by mammography before NACT | Removed (LASSO) |
|  | Yes |  |  |
| MAMMO MF/MC preNACT | No | Presence of multifocality or multicentricity, evaluated by mammography before NACT | Removed (high correlation) |
|  | Yes |  |  |
| MAMMO microcalcification preNACT | No | Presence of microcalcifications associated with the tumor, evaluated by mammography before NACT | Removed (LASSO) |
|  | Yes |  |  |
| MAMMO suspect LN preNACT | No | Presence of suspicious lymph nodes, evaluated by mammography before NACT | Removed (LASSO) |
|  | Yes |  |  |
| MAMMO number suspect LN preNACT |  | Number of suspect lymph nodes, evaluated by mammography before NACT | Removed (high correlation) |
| Breast density ACR | I | Breast density based on ACR classification | Included |
|  | II |  |  |
|  | III |  |  |
|  | IV |  |  |
| MRI largest DIA preNACT |  | Largest diameter of the tumor in mm, evaluated by MRI before NACT | Removed (missing values) |
| MRI multifocality preNACT | No | Presence of multifocality, evaluated by MRI before NACT | Removed (missing values) |
|  | Yes |  |  |
| MRI multicentricity preNACT | No | Presence of multicentricity, evaluated by MRI before NACT | Removed (missing values) |
|  | Yes |  |  |
| MRI MF/MC preNACT | No | Presence of multifocality or multicentricity, evaluated by MRI before NACT | Removed (missing values) |
|  | Yes |  |  |
| MRI suspect LN preNACT | No | Presence of suspicious lymph nodes, evaluated by MRI before NACT | Removed (missing values) |
|  | Yes |  |  |
| MRI number suspect LN preNACT |  | Number of suspect lymph nodes, evaluated by MRI before NACT | Removed (missing values) |
| Palpability NACT1 | No | Palpability in physical examination after first NACT cycles | Removed (LASSO) |
|  | Yes |  |  |
| US largest DIA NACT1 |  | Largest diameter of the tumor in mm, evaluated by ultrasound after first NACT cycles | Included |
| US multifocality NACT1 | No | Presence of multifocality, evaluated by ultrasound after first NACT cycles | Removed (LASSO) |
|  | Yes |  |  |
| US multicentricity NACT1 | No | Presence of multicentricity, evaluated by ultrasound after first NACT cycles | Removed (LASSO) |
|  | Yes |  |  |
| US MF/MC NACT1 | No | Presence of multifocality or multicentricity, evaluated by ultrasound after first NACT cycles | Removed (high correlation) |
|  | Yes |  |  |
| US suspect LN NACT1 | No | Presence of suspicious lymph nodes, evaluated by ultrasound after first NACT cycles | Removed (LASSO) |
|  | Yes |  |  |
| US number suspect LN NACT1 |  | Number of suspect lymph nodes, evaluated by ultrasound after first NACT cycles | Removed (LASSO) |
| MAMMO largest DIA NACT1 |  | Largest diameter of the tumor in mm, evaluated by mammography after first NACT cycles | Included |
| MAMMO multifocality NACT1 | No | Presence of multifocality, evaluated by mammography after first NACT cycles | Removed (LASSO) |
|  | Yes |  |  |
| MAMMO multicentricity NACT1 | No | Presence of multicentricity, evaluated by mammography after first NACT cycles | Removed (LASSO) |
|  | Yes |  |  |
| MAMMO MF/MC NACT1 | No | Presence of multifocality or multicentricity, evaluated by mammography after first NACT cycles | Removed (high correlation) |
|  | Yes |  |  |
| MAMMO microcalcification NACT1 | No | Presence of microcalcifications associated with the tumor, evaluated by mammography after first NACT cycles | Removed (LASSO) |
|  | Yes |  |  |
| MAMMO suspect LN NACT1 | No | Presence of suspicious lymph nodes, evaluated by mammography after first NACT cycles | Removed (LASSO) |
|  | Yes |  |  |
| MAMMO number suspect LN NACT1 |  | Number of suspect lymph nodes, evaluated by mammography after first NACT cycles | Removed (high correlation) |
| MRI largest DIA NACT1 |  | Largest diameter of the tumor in mm, evaluated by MRI after first NACT cycles | Removed (missing values) |
| MRI multifocality NACT1 | No | Presence of multifocality, evaluated by MRI after first NACT cycles | Removed (missing values) |
|  | Yes |  |  |
| MRI multicentricity NACT1 | No | Presence of multicentricity, evaluated by MRI after first NACT cycles | Removed (missing values) |
|  | Yes |  |  |
| MRI MF/MC NACT1 | No | Presence of multifocality or multicentricity, evaluated by MRI after first NACT cycles | Removed (missing values) |
|  | Yes |  |  |
| MRI suspect LN NACT1 | No | Presence of suspicious lymph nodes, evaluated by MRI after first NACT cycles | Removed (missing values) |
|  | Yes |  |  |
| MRI number suspect LN NACT1 |  | Number of suspect lymph nodes, evaluated by MRI after first NACT cycles | Removed (missing values) |
| Palpability NACT2 | No | Palpability in physical examination after second NACT cycles | Removed (missing values) |
|  | Yes |  |  |
| US largest DIA NACT2 |  | Largest diameter of the tumor in mm, evaluated by ultrasound after second NACT cycles | Removed (missing values) |
| US multifocality NACT2 | No | Presence of multifocality, evaluated by ultrasound after second NACT cycles | Removed (missing values) |
|  | Yes |  |  |
| US multicentricity NACT2 | No | Presence of multicentricity, evaluated by ultrasound after second NACT cycles | Removed (missing values) |
|  | Yes |  |  |
| US MF/MC NACT2 | No | Presence of multifocality or multicentricity, evaluated by ultrasound after second NACT cycles | Removed (missing values) |
|  | Yes |  |  |
| US suspect LN NACT2 | No | Presence of suspicious lymph nodes, evaluated by ultrasound after second NACT cycles | Removed (missing values) |
|  | Yes |  |  |
| US number suspect LN NACT2 |  | Number of suspect lymph nodes, evaluated by ultrasound after second NACT cycles | Removed (missing values) |
| MAMMO largest DIA NACT2 |  | Largest diameter of the tumor in mm, evaluated by mammography after second NACT cycles | Removed (missing values) |
| MAMMO multifocality NACT2 | No | Presence of multifocality, evaluated by mammography after second NACT cycles | Removed (missing values) |
|  | Yes |  |  |
| MAMMO multicentricity NACT2 | No | Presence of multicentricity, evaluated by mammography after second NACT cycles | Removed (missing values) |
|  | Yes |  |  |
| MAMMO MF/MC NACT2 | No | Presence of multifocality or multicentricity, evaluated by mammography after second NACT cycles | Removed (missing values) |
|  | Yes |  |  |
| MAMMO microcalcification NACT2 | No | Presence of microcalcifications associated with the tumor, evaluated by mammography after second NACT cycles | Removed (missing values) |
|  | Yes |  |  |
| MAMMO suspect LN NACT2 | No | Presence of suspicious lymph nodes, evaluated by mammography after second NACT cycles | Removed (missing values) |
|  | Yes |  |  |
| MAMMO number suspect LN NACT2 |  | Number of suspect lymph nodes, evaluated by mammography after second NACT cycles | Removed (missing values) |
| MRI largest DIA NACT2 |  | Largest diameter of the tumor, evaluated by MRI after second NACT cycles | Removed (missing values) |
| MRI multifocality NACT2 | No | Presence of multifocality, evaluated by MRI after second NACT cycles | Removed (missing values) |
|  | Yes |  |  |
| MRI multicentricity NACT2 | No | Presence of multicentricity, evaluated by MRI after second NACT cycles | Removed (missing values) |
|  | Yes |  |  |
| MRI MF/MC NACT2 | No | Presence of multifocality or multicentricity, evaluated by MRI after second NACT cycles | Removed (missing values) |
|  | Yes |  |  |
| MRI suspect LN NACT2 | No | Presence of suspicious lymph nodes, evaluated by MRI after second NACT cycles | Removed (missing values) |
|  | Yes |  |  |
| MRI number suspect LN NACT2 |  | Number of suspect lymph nodes, evaluated by MRI after second NACT cycles | Removed (missing values) |
| Palpability postNACT | No | Palpability in physical examination after NACT | Included |
|  | Yes |  |  |
| US largest DIA postNACT |  | Largest diameter of the tumor in mm, evaluated by ultrasound after NACT | Removed (LASSO) |
| US multifocality postNACT | No | Presence of multifocality, evaluated by ultrasound after NACT | Removed (high correlation) |
|  | Yes |  |  |
| US multicentricity postNACT | No | Presence of multicentricity, evaluated by ultrasound after NACT | Removed (LASSO) |
|  | Yes |  |  |
| US MF/MC postNACT | No | Presence of multifocality or multicentricity, evaluated by ultrasound after NACT | Removed (LASSO) |
|  | Yes |  |  |
| US suspect LN postNACT | No | Presence of suspicious lymph nodes, evaluated by ultrasound after NACT | Removed (LASSO) |
|  | Yes |  |  |
| US number suspect LN postNACT |  | Number of suspect lymph nodes, evaluated by ultrasound after NACT | Removed (LASSO) |
| US ycT stage | ycT0 (cCR) | ycT stage, evaluated by ultrasound after NACT (ycTnear: suspicious findings without clearly delineable lesion, ycTX: lack of clear description in radiology reports) | Removed (LASSO) |
|  | ycTis |  |  |
|  | ycT1mic |  |  |
|  | ycT1a |  |  |
|  | ycT1b |  |  |
|  | ycT1c |  |  |
|  | ycT2 |  |  |
|  | ycT3 |  |  |
|  | ycT4 |  |  |
|  | ycTnear |  |  |
|  | ycTX |  |  |
| US ycN stage | ycN0 | ycN stage, evaluated by ultrasound after NACT | Removed (LASSO) |
|  | ycN+ |  |  |
| US assessment postNACT | cCR | Assessment of ultrasound after NACT (based on the US ycT stage, ycTX cases were considered as missing values) | Included |
|  | Near-cCR |  |  |
|  | Non-cCR |  |  |
| MAMMO largest DIA postNACT |  | Largest diameter of the tumor in mm, evaluated by mammography after NACT | Removed (LASSO) |
| MAMMO multifocality postNACT | No | Presence of multifocality, evaluated by mammography after NACT | Removed (LASSO) |
|  | Yes |  |  |
| MAMMO multicentricity postNACT | No | Presence of multicentricity, evaluated by mammography after NACT | Removed (LASSO) |
|  | Yes |  |  |
| MAMMO MF/MC postNACT | No | Presence of multifocality or multicentricity, evaluated by mammography after NACT | Removed (high correlation) |
|  | Yes |  |  |
| MAMMO microcalcification postNACT | No | Presence of microcalcifications associated to the tumor, evaluated by mammography after NACT | Removed (LASSO) |
|  | Yes |  |  |
| MAMMO suspect LN postNACT | No | Presence of suspicious lymph nodes, evaluated by mammography after NACT | Removed (LASSO) |
|  | Yes |  |  |
| MAMMO number suspect LN postNACT |  | Number of suspect lymph nodes, evaluated by mammography after NACT | Removed (LASSO) |
| MAMMO ycT stage | ycT0 (cCR) | ycT stage, evaluated by mammography after NACT (ycTnear: suspicious findings without clearly delineable lesion, ycTX: lack of clear description in radiology reports) | Removed (LASSO) |
|  | ycTis |  |  |
|  | ycT1mic |  |  |
|  | ycT1a |  |  |
|  | ycT1b |  |  |
|  | ycT1c |  |  |
|  | ycT2 |  |  |
|  | ycT3 |  |  |
|  | ycT4 |  |  |
|  | ycTnear |  |  |
|  | ycTX |  |  |
| MAMMO ycN stage | ycN0 | ycN stage, evaluated by mammography after NACT | Removed (LASSO) |
|  | ycN+ |  |  |
| MAMMO assessment postNACT | cCR | Assessment of mammography after NACT (based on the MAMMO ycT stage, ycTX cases were considered as missing values) | Included |
|  | Near-cCR |  |  |
|  | Non-cCR |  |  |
| MRI largest DIA postNACT |  | Largest diameter of the tumor in mm, evaluated by MRI after NACT | Removed (missing values) |
| MRI multifocality postNACT | No | Presence of multifocality, evaluated by MRI after NACT | Removed (missing values) |
|  | Yes |  |  |
| MRI multicentricity postNACT | No | Presence of multicentricity, evaluated by MRI after NACT | Removed (missing values) |
|  | Yes |  |  |
| MRI MF/MC postNACT | No | Presence of multifocality or multicentricity, evaluated by MRI after NACT | Removed (missing values) |
|  | Yes |  |  |
| MRI suspect LN postNACT | No | Presence of suspicious lymph nodes, evaluated by MRI after NACT | Removed (missing values) |
|  | Yes |  |  |
| MRI number suspect LN postNACT |  | Number of suspect lymph nodes, evaluated by MRI after NACT | Removed (missing values) |
| MRI ycT stage | ycT0 (cCR) | ycT stage, evaluated by MRI after NACT (ycTnear: suspicious findings without clearly delineable lesion, ycTX: lack of clear description in radiology reports) | Removed (missing values) |
|  | ycTis |  |  |
|  | ycT1mic |  |  |
|  | ycT1a |  |  |
|  | ycT1b |  |  |
|  | ycT1c |  |  |
|  | ycT2 |  |  |
|  | ycT3 |  |  |
|  | ycT4 |  |  |
|  | ycTnear |  |  |
|  | ycTX |  |  |
| MRI ycN stage | ycN0 | ycN stage, evaluated by MRI after NACT | Removed (missing values) |
|  | ycN+ |  |  |
| MRI assessment postNACT | cCR | Assessment of MRI after NACT (based on the MRI ycT stage, ycTX cases were considered as missing values) | Removed (missing values) |
|  | Near-cCR |  |  |
|  | Non-cCR |  |  |
| US/MAMMO ycT stage | ycT0 (cCR) | ycT-stage, based on combined evaluation of ultrasound and mammography after NACT | Removed (LASSO) |
|  | ycTis |  |  |
|  | ycT1mic |  |  |
|  | ycT1a |  |  |
|  | ycT1b |  |  |
|  | ycT1c |  |  |
|  | ycT2 |  |  |
|  | ycT3 |  |  |
|  | ycT4 |  |  |
|  | ycTnear |  |  |
|  | ycTX |  |  |
| US/MAMMO ycN stage | ycN0 | ycN stage, based on combined evaluation of ultrasound and mammography after NACT | Removed (LASSO) |
|  | ycN+ |  |  |
| US/MAMMO clinical remission status | cCR | Assessment of ultrasound and mammography after NACT (based on the US/MAMMO ycT stage) | Removed (LASSO) |
|  | Near-cCR |  |  |
|  | Non-cCR |  |  |
| US/MAMMO/MRI ycT stage | ycT0 (cCR) | ycT-stage, based on combined evaluation of ultrasound, mammography and MRI after NACT | Included |
|  | ycTis |  |  |
|  | ycT1mic |  |  |
|  | ycT1a |  |  |
|  | ycT1b |  |  |
|  | ycT1c |  |  |
|  | ycT2 |  |  |
|  | ycT3 |  |  |
|  | ycT4 |  |  |
|  | ycTnear |  |  |
|  | ycTX |  |  |
| US/MAMMO/MRI ycN stage | ycN0 | ycN stage, based on combined evaluation of ultrasound, mammography and MRI after NACT | Removed (LASSO) |
|  | ycN+ |  |  |
| US/MAMMO/MRI clinical remission status | cCR | Assessment of ultrasound, mammography and MRI after NACT (based on the US/MAMMO/MRI ycT stage) | Removed (LASSO) |
|  | Near-cCR |  |  |
|  | Non-cCR |  |  |
| Time to OR |  | Time interval between postNACT examination and surgical resection, expressed in days | Removed (LASSO) |
| Outcome | Benign | Results from the histopathological examination | Outcome variable of the model |
|  | Malignant |  |  |

Legend: Removed (LASSO), removed due to LASSO regularization, 48 variables; Removed (missing values), removed due to ≥ 50 % missing values, 41 variables; Removed (high correlation), removed due to > 90% correlation with another variable, 8 variables; Included, included in the final GLM model, 14 variables.

2) Recipe for the pre-processing of variables prior to LASSO regularization and model training:

- step_impute_knn(all_predictors(), neighbors = 5) %>%
- step_BoxCox(all_numeric(),-all_outcomes()) %>%
- step_other(all_nominal(), threshold = .05, other = "other") %>%
- step_zv(all_predictors(),-all_outcomes()) %>%
- step_nzv(all_predictors(),-all_outcomes())%>%
- step_normalize(all_numeric(),-all_outcomes())%>%
- step_dummy(all_nominal(),-all_outcomes()) %>%
- step_corr(all_predictors(),-all_outcomes(), threshold = 0.9)

3) Performance Metrics of additional models:

XGboost (Extreme Gradient Boosting tree)

| **Performance Metric** | **Development Set** | **Validation Set** |
| --- | --- | --- |
| False Positive Rate | 4.9 % (27 of 552) | 10.9 % (5 of 46) |
| Positive Predictive Value | 82.4 % (126 of 153) | 66.7 % (10 of 15) |
| Accuracy | 69.8 % | 66.2 % |
| No Information Rate | 59.2 % | 59.7 % |
| P-Value (ACC > NIR) | < 0.001 | 0.148 |
| AUC | 0.805 (0.779 to 0.831) | 0.792 (0.652 to 0.874) |

MARS algorithm (Multivariate Adaptive Regression Spline)

| **Performance Metric** | **Development Set** | **Validation Set** |
| --- | --- | --- |
| False Positive Rate | 4.9 % (27 of 552) | 10.9 % (5 of 46) |
| Positive Predictive Value | 77.9 % (95 of 122) | 68.8 % (11 of 16) |
| Accuracy | 66.5% | 67.5 % |
| No Information Rate | 59.2% | 59.7 % |
| P-Value (ACC > NIR) | < 0.001 | 0.100 |
| AUC | 0.788 (0.761 to 0.815) | 0.796 (0.690 to 0.891) |

SVM (Support Vector Machine with polynomial kernel)

| **Performance Metric** | **Development Set** | **Validation Set** |
| --- | --- | --- |
| False Positive Rate | 4.9 % (27 of 552) | 10.9 % (5 of 46) |
| Positive Predictive Value | 81.8 % (121 of 148) | 66.7 % (10 of 15) |
| Accuracy | 69.2 % | 66.2 % |
| No Information Rate | 59.2 % | 59.7 % |
| P-Value (ACC > NIR) | < 0.001 | 0.148 |
| AUC | 0.809 (0.781 to 836) | 0.770 (0.665 to 0.865) |
